# Distribution and the origin of invasive apple snails, *Pomacea canaliculata* and *P. maculata* (Gastropoda: Ampullariidae) in China

**DOI:** 10.1038/s41598-017-19000-7

**Published:** 2018-01-19

**Authors:** Qian-Qian Yang, Su-Wen Liu, Chao He, Xiao-Ping Yu

**Affiliations:** Zhejiang Provincial Key Laboratory of Biometrology and Inspection and Quarantine, College of Life Sciences, China Jiliang University, Hangzhou, 310018 China

## Abstract

Species of *Pomacea*, commonly known as apple snails, are native to South America, and have become widely distributed agricultural and environmental pests in southern China since their introduction in the 1980s. However, only since 2010 have researchers recognized that at least two species, *P. canaliculata* and *P. maculata*, are present in China. Although impacts of apple snails have been extensively documented, confusion still persists regarding current distributions and origin of the species in China. To resolve this confusion, we used phylogenetic and phylogeographic methods to analyze 1464 mitochondrial COI sequences, including 349 new sequences from samples collected in southern China and 1115 publicly available sequences from snails collected in the native and introduced ranges. *Pomacea canaliculata* was found at all sampled localities, while *P. maculata* was found at only five sampled localities in the Sichuan basin and Zhejiang province. Our data indicate that Chinese populations of *P. canaliculata* share an Argentinian origin, consistent with multiple introductions of this species elsewhere in Asia. In addition, just a single lineage of *P. maculata* is established in China, which shares with populations in Brazil.

## Introduction

Apple snails (Ampullariidae), are freshwater gastropods native to South America^1^, and several species in the genus *Pomacea* have been introduced and become established in many parts of the world including other Asian countries, North America, islands of the Pacific, and Europe^2,3^. They have a voracious appetite^4,5^, reproduce rapidly^6^, are resistant to desiccation during dry down periods^7^, and act as vectors of zoonotic diseases^8^, all of which have made them serious agricultural^9^, environmental^1^, and potential human health pests^10^.

With its highly diverse biogeography, topography, and climate, China offers numerous opportunities for a range of invasive species, and those that have been introduced have impacted China significantly^11^. Apple snails were initially introduced to the mainland of China, from Taiwan to Zhongshan city, Guangdong province, in 1981 for aquaculture^12,13^. The first ten years after introduction saw a rapid expansion of the range of apple snails in China, with a boom in aquaculture and economic interests as the main driver^12^. In the early 1980s, apple snails were introduced to at least 18 cities in11 provinces/municipalities, including Guangdong, Guangxi, Fujian, Zhejiang, Jiangxi, Jiangsu, Shanghai, Anhui, Hubei in the south, and Beijing and Liaoning in the north. In the mid-1980s, another 12 cities reported introduction of apple snails, including the southern provinces of Zhejiang, Yunnan, Sichuan, Chongqing and Jiangxi, and the northern provinces of Gansu and Tianjin^12^. Intentional introductions declined sharply after the 1990s, because of the poor market benefits and realization of significant crop damage caused by the snails. The spread also slowed, with limited expansion due to unintentional human transport and natural diffusion^12^.

*Pomacea canaliculata* (Lamarck, 1822) and *P. maculata* Perry, 1810 are the two most common and highly invasive apple snail species^14^. However, many other alien apple snail species were difficult to differentiate from *P. canaliculata* and *P. maculata*, which were frequently misidentified as these two species^3,15,16^. Additionally, for a long time, *P. canaliculata* was presumed to be the only alien apple snail species in Asia and was listed as one of 100 of the world’s worst invasive alien species^17^. However, Hayes *et al*., using a combination of morphological and DNA sequence data recognized four species of *Pomacea* as having been introduced into Asia^3^. Subsequently, Hayes *et al*. provided clear anatomical and biogeographic data for delineating between these two previously conflated species^14^.

In Asia, *P. canaliculata* was introduced to Asia more than once from multiple locations in Argentina, while *P. maculata* was introduced to Asia from Brazil and Argentina independently^3^. However, only 5 samples of *P. canaliculata* was recorded in China by Hayes *et al*.^3^. Subsequently, Song *et al*. and Lv *et al*. reported that both species, *P. canaliculata* and *P. maculata*, were established in China^13,18^. However, after the distribution pattern of apple snails sampled in 2006 and 2007 in China by Lv *et al*.^13^, there are no tracking updates of their spread until now. In this study, we combined phylogenetic and phylogeographic analyses of mtDNA COI sequences of *P. canaliculata* and *P. maculata* collected from across their range in China to fully document their origin and current distributions in China.

## Material and Methods

### Sample collection and DNA extraction

August 2014 – July 2015, we surveyed 34 localities in 14 provinces in mainland China in which apple snails might occur, collecting 44 adults and 305 egg masses from 31 locations in 12 provinces (Table 1). Subsamples of foot tissues and eggs, from each sampled population, were preserved in 100% ethanol and stored at −20 °C prior to extraction of DNA.

**Table 1:** Sampling information for Chinese apple snails sequenced in this study. *N* represents number of sequenced samples. Only accession number represented unique haplotype were showed in the table.

Sample code	*N*	Locality	Habitat	Haplotype identified (GenBank accession number)
HNHK	4	Meilan, Haikou, Hainan	Pond	PcH1 (KP310264), PcH7 (KT852757)
GDGZ	17	Tianhe, Guangzhou, Guangdong	Paddy	PcH1 (KP310264), PcH2 (KP310375)
JYBY	6	Banyang, Jieyang, Guangdong	Pond	PcH1 (KP310264), PcH2 (KP310375), PcH5 (KP310439)
JYTP	9	Tangpu, Jieyang, Guangdong	Paddy	PcH1 (KP310264)
GXNN	4	Xixiangtang, Nanning, Guangxi	Pond	PcH2 (KP310375)
GXWZ	17	Shiqiao, Wuzhou, Guangxi	River	PcH1 (KP310264), PcH5 (KP310439)
HZBB	9	Babu, Hezhou, Guangxi	Pond	PcH1 (KP310264), PcH2 (KP310375)
GLBS	9	Baisha, Guilin, Guangxi	Pond	PcH1 (KP310264)
GLYS	8	Yangsu, Guilin, Guangxi	Pond	PcH1 (KP310264)
YNKM	4	Guandu, Kunming, Yunnan	Paddy	PcH2 (KP310375)
YNDL	7	Erhai, Dali,Yunnan	Lake	PcH1 (KP310264), PcH2 (KP310375)
FJXM	22	Tongan, Xiamen, Fujian	Pond	PcH1 (KP310264), PcH2 (KP310375)
FJFZ	8	Cangshan, Fuzhou, Fujian	Paddy	PcH2 (KP310375)
JXGZ	12	Xinfeng, Ganzhou, Jiangxi	Pond	PcH1 (KP310264), PcH2 (KP310375), PcH4 (KP310443)
JXSR	15	Xinzhou, Shangrao, Jiangxi	Pond	PcH1 (KP310264), PcH2 (KP310375)
LYFY	15	Fengyu, Liuyang, Hunan	Paddy	PcH1 (KP310264)
CSQY	2	Qiaoyi, Changsha, Hunan	River	PcH2 (KP310375)
GZGY	8	Nanming, Guiyang, Guizhou	Paddy	PcH1 (KP310264)
WZLC	12	Lucheng, Wenzhou, Zhejiang	Paddy	PcH1 (KP310264), PcH2 (KP310375), PcH5 (KP310439)
ZJSX	9	Xinchang, Shaoxing, Zhejiang	Pond	PcH2 (KP310375), PcH3 (KR021020)
ZJZS	34	Putuo, Zhoushan, Zhejiang	Paddy	PcH1 (KP310264), PcH2 (KP310375)
ZJYY	3	Yuyao, Ningbo, Zhejiang	Paddy	PcH1 (KP310264), PcH4 (KP310443)
ZJSY	7	Shangyu, Shaoxing, Zhejiang	Pond	PcH2 (KP310375), PcH3 (KR021020)
HZACA	14	Jianggan, Hangzhou, Zhejiang	Lake	PcH2 (KP310375), PmH1 (KT852782), PmH2 (KT852786)
HZXH	10	Xihu, Hangzhou, Zhejiang	Lake	PcH1 (KP310264), PcH2 (KP310375)
CQHY	12	Huayan, Shapingba, Chongqing	Lotus pond	PcH1 (KP310264), PcH2 (KP310375), PmH1 (KT852782)
CQHC	10	Hechuan, Chongqing	Lotus pond	PcH2 (KP310375), PcH5 (KP310439), PmH1 (KT852782)
SCSN	18	Xiwu Wetland, Suining, Sichuan	Wetland pond	PcH1 (KP310264), PcH2 (KP310375), PmH1 (KT852782)
CDJJ	12	Jinjiang, Chengdu, Sichuan	Lotus pond	PcH1 (KP310264), PcH2 (KP310375), PmH1 (KT852782)
JSWJ	11	WuJiang, Suzhou, Jiangsu	River	PcH2 (KP310375), PcH6 (KP310290)
JSWZ	21	Wuzhong, Suzhou, Jiangsu	Pond	PcH2 (KP310375)

Genomic DNA was extracted from approximately 10 mg of foot tissue or a single egg from each clutch using the DNeasy Blood and Tissue Extraction Kit (QIAGEN) following the manufacturers’ protocol, with final elution of 200 µL. Eggs from each clutch were separated using a 10% sodium hydroxide solution^19^.

### Amplification and sequencing

A portion of the mitochondrial gene cytochrome *c* oxidase subunit I (COI) was amplified using the primers LCO1490 and HCO2198^20^ in 25 µL reactions containing 0.625 U TaKaRa *Ex* Taq, 1 × *Ex* Taq Buffer, 5 mM dNTP mixture, 10 µM of each primer and 1 µL of genomic DNA. Cycle conditions consisted of an initial denaturation for 3 min at 95 °C, followed by 35 cycles of 30 s at 95 °C, 30 s at 50 °C, and 1 min at 72 °C, followed by a final extension step of 72 °C for 8 min and 10 min at 4 °C. Amplicons were visualized and checked for specificity via gel electrophoresis and single product amplicons were sent to Sunny Biotechnology (Shanghai, China) for sequencing in both directions. All sequences were checked for errors and edited manually in Chromas 1.0^21^. We finally obtained 349 COI sequences of 657 bp. Species were prior distinguished through phylogenetic analyses, and then all sequences were deposited in GenBank (Table 1).

### COI datasets

We added 607 COI sequences from Hayes *et al*. to the 349 COI sequences generated in this study^3^. The sequences from Hayes *et al*. included sequences from the native and introduced ranges, with 466 sequences of *P. canaliculata*, 18 from China (five from the mainland and 13 from Taiwan), and 141 sequences of *P. maculata*^3^.

We downloaded another 151 sequences from GenBank from other studies, excluding those from Hayes *et al*.^3^. However, given the widespread issues with misidentification of *Pomacea* species, we filtered COI sequences from GenBank using the following criteria: (1) sequences were published after 2007 when it became possible to distinguish *P. maculata* sequences from those of *P. canaliculata*^2^; (2) sequences were verified as being correctly identified through phylogenetic systematic approaches (see below). After filtering, we discarded 33 sequences and added 118 to our matrix, including 52 sequences of *P. canaliculata*, and 66 sequences of *P. maculata* (Supplementary Table S1). We also added 390 sequences from Lv *et al*. Appendix S1, including 389 from *P. canaliculata* and one sequence of *P. maculata* (Table 1 of Lv *et al*.)^13^.

### Phylogenetic analyses

The total matrix consisted of 1464 sequences that varied in length from 503 bp^13^ to 657 bp (this study). We added COI sequences from *P. lineata* (FJ710310)^22^ and *P. paludosa* (EU528477)^3^ to serve as outgroups. All sequences were assembled and aligned in ClustalW implemented in MEGA 6.0^23^. The best sequence substitution model (GTR + I + G) for the data set was selected using the AIC in jModelTest ver. 2.1.7^24^. Phylogenetic relationships among all COI sequences was reconstructed under Maximum Likelihood implemented in MEGA 6.0 with node support assessed using 1000 bootstrap replicates^23,25^.

### Haplotype distribution and network analyses

Clades containing both *P. maculata* and *P. canaliculata* were identified from the phylogenetic analyses based on COI sequences, and these sequences were used to created haplotype networks in TCS 1.21 for each species^26^. The parsimony connection limit for haplotype network reconstruction was set to 95% for all analyses. We also mapped haplotype distributions in China using ArcGIS 10.2.

Prior to analyses unique haplotypes for each species were identified in DnaSP 5.1^27^. Because the sequence lengths were different, prior to haplotype analysis three datasets consisting of only *P. canaliculata* and *P. maculata* haplotypes were constructed. (1) sequences from our study only (Dataset 1), 2) sequences from our study, plus those of Hayes *et al*., and those filtered from GenBank (Dataset 2), and 3) dataset 2 plus Lv *et al*. sequences (Dataset 3)^3,13^. Datasets were created by trimming all sequences to the shortest length for each species. Dataset 1 was 657 bp for both species, Dataset 2 was 558 bp for *P. canaliculata* and 577 bp *P. maculata*, and Dataset 3 was 503 bp for both.

### Mismatch distribution analyses

Introduction scenarios and signals of historical population expansion was examined with mismatch analyses^28,29^. Theoretically, a mismatch distribution analyses for populations after bottlenecks followed by sudden expansions should generate well-separated peak patterns for each population, with each unique introduction source generating a separate peak^3^.

Patterns of genetic variation for *P. canaliculata* and *P. maculata* in mainland China were examined based on mismatch distribution analyses. We conducted the mismatch distribution analyses by comparing the number of pairwise differences at all sites of the COI sequences using DnaSP 5.1.

### Data accessibility

All sequences were submitted in GenBank under accession numbers KP310264-KP310445, KP310474, KP310480-KP310496, KR020942-KR021020, KR021027, KR021034-KR021040, KT852706-KT852762, and KT852782-KT852786.

## Results

### Phylogenetic systematics

Apple snails were found at 31 of the 34 sites surveyed. Phylogenetic analyses recovered all sequences from these newly collected samples and all others on mainland China in two well supported, monophyletic clades. Of the 1464 COI sequences, 1226 were recovered in a clade identified as *P. canaliculata*, and the remaining 238 sequences were *P. maculata* (Fig. S1).

### Haplotype distribution in China

There were no appreciable differences in the results derived from the three different datasets of different lengths, as such we only report the results from Dataset 3, which contained all sequences trimmed to 503 bp. From this dataset, the 1226 *P. canaliculata* sequences produced 58 unique haplotypes (PcH1- PcH58), while there were only 37 unique haplotypes from *P. maculata* (PmH1-PmH37; Table 2).

**Table 2:** Haplotype distributions of countries/regions included in this study.

Continent	Country/Region	No. of sequences (PcH/PmH)	No. of haplotypes (PcH/PmH)	Haplotype distribution
Asia	China-mainland	718/32	23/3	PcH1 (**159 + **254), PcH2 (**129 + **75), PcH3 (**14** + 1), PcH4 (**5 + **35), PcH5 (**10 + **16), PcH6 (**1**), PcH7 (**1**), PcH43 (1), PcH44 (1), PcH45 (1), PcH46 (1), PcH47 (1), PcH48 (1), PcH49 (1), PcH50 (1), PcH51 (1), PcH52 (1), PcH53 (3), PcH54 (1), PcH55 (1), PcH56 (1), PcH57 (1), PcH58 (1), PmH1 (**29 + **1), PmH2 (**1**), PmH37 (1)
	China-Taiwan	13/0	4/0	PcH2 (8), PcH6 (3), PcH36 (1), PcH37 (1)
	Japan	25/6	7/2	PcH1 (1), PcH2 (7), PcH3 (3), PcH5 (4), PcH6 (5), PcH11 (3), PcH36 (2), PmH1 (3), PmH3 (3)
	Philippines	266/0	13/0	PcH2 (187), PcH4 (36), PcH5 (15), PcH11 (15), PcH25 (1), PcH26 (1), PcH27 (1), PcH28 (1), PcH29 (4), PcH30 (2), PcH31 (1), PcH32 (1), PcH33 (1)
	Vietnam	10/11	2/1	PcH2 (6), PcH5 (4), PmH1 (11)
	Thailand	2/17	2/2	PcH34 (1), PcH35 (1), PmH1 (11), PmH3 (6)
	Myanmar	9/1	2/1	PcH1 (4), PcH2 (5), PmH1 (1)
	Indonesia	6/0	2/0	PcH22 (3), PcH23 (3)
	Korea	12/1	2/1	PcH2 (9), PcH6 (3), PmH5 (1)
	Laos	2/0	1/0	PcH5 (2)
	Singapore	0/4	0/1	PmH1 (4)
	Cambodia	0/5	0/1	PmH1 (5)
Oceania	Papua New Guinea	6/0	2/0	PcH2 (3), PcH24 (3)
Europe	Spain	0/7	0/1	PmH1 (7)
	Belgium	0/5	0/1	PmH1 (5)
North America	USA	58/53	1/3	PcH2 (58), PmH1 (10), PmH3 (20), PmH35 (23)
South America	Uruguay	9/0	1/0	PcH38 (9)
	Brazil	0/60	0/26	PmH1 (1), PmH10 (8), PmH11 (2), PmH12 (1), PmH13 (1), PmH14 (4), PmH15 (5), PmH16 (5), PmH17 (1), PmH18 (6), PmH19 (2), PmH20 (1), PmH21 (2), PmH22 (1), PmH23 (6), PmH24 (1), PmH25 (1), PmH26 (2), PmH27 (1), PmH28 (1), PmH29 (1), PmH30 (2), PmH31 (1), PmH32 (1), PmH33 (1), PmH34 (2)
	Argentina	90/36	19/8	PcH2 (47), PcH8 (1), PcH9 (1), PcH10 (1), PcH11 (7), PcH12 (4), PcH13 (3), PcH14 (3), PcH15 (4), PcH16 (5), PcH17 (1), PcH18 (1), PcH19 (2), PcH20 (2), PcH21 (1), PcH39 (1), PcH40 (3), PcH41 (1), PcH42 (1), PmH3 (2), PmH4 (1), PmH5 (20), PmH6 (8), PmH7 (2), PmH8 (1), PmH9 (1), PmH36 (1)

PcH and PmH represent haplotypes for *P. canaliculata* and *P. maculata*, respectively. All haplotypes were identified using Database 3. Numbers in bold represent 349 apple snails sampled in our study.

**Figure 1:**
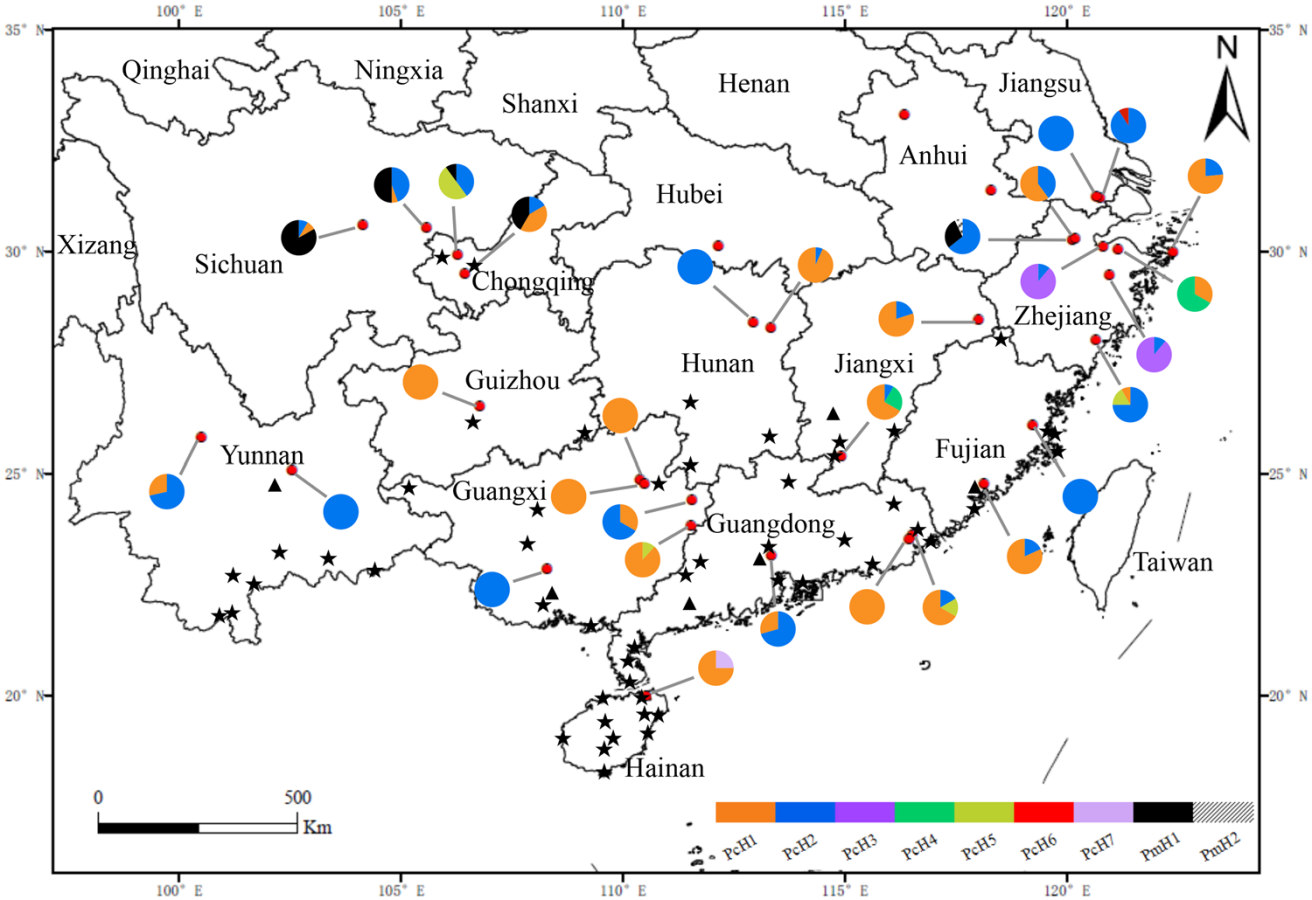
Geographical distribution and frequency of *P. canaliculata* (PcH) and *P. maculata* (PmH) haplotypes in China. The map was created in ArcGIS 10.2 software (ESRI Inc., Redlands, CA, USA). URL http://www.esri.com/software/arcgis/arcgis-for-desktop. Red circles indicate survey sites with snails sampled during this study, and colors of the associated pie charts represent haplotype frequencies at each site. Stars indicate localities sampled by Lv *et al*.^13^ and triangles represent localities from Song *et al*.^18^.

Seven *P. canaliculata* haplotypes representing 319 sequences (PcH1~PcH7) and two *P. maculata* haplotypes representing 30 sequences (PmH1and PmH2) were recovered. *Pomacea canaliculata* was found at all 31 sites, and *P. maculata* haplotypes were recorded from only five populations in the Sichuan province, Chongqing municipality, and Zhejiang province (Fig. 1). Twenty-two (71%) of the populations contained multiple haplotypes, and nine only had a single haplotype (four with only PcH1 and five with PcH2; Fig. 1).

Among the *P. canaliculata* haplotypes, PcH2 was the most widely distributed geographically (24 sites), accounting for 40% (*n* = 129) of the snails. Snails with this haplotype were mainly distributed along the eastern and southern coastal regions and at the northern edge of the range (Fig. 1). The second most widely distributed haplotype, PcH1, was detected in 21 sites, but made up 50% of the snails, which were primarily collected in the rural interior (Fig. 1). The remaining haplotypes, PcH3, PcH4, and PcH5 came from 5–14 sequences each, and were less widely distributed, found in two populations of Zhejiang province, two populations of Zhejiang and Jiangxi provinces, and four populations of Zhejiang, Guangdong, Guangxi, and Chongqing provinces, respectively. Two haplotypes, PcH6 and PcH7, were represented by a single sequence each and were found in only one population each, in Jiangsu and Hainan provinces, respectively (Fig. 1). Two haplotypes were recovered from the 30 *P. maculata* sequences, with PmH1 represented by 29 sequences occurring in five populations of Sichuan, Chongqing, and Zhejiang provinces. The other haplotype, PmH2, was represented by only one sequence in Zhejiang province (Fig. 1). Together, Dataset 3 (all sequences) produced 25 *P. canaliculata* haplotypes and 3 *P. maculata* haplotypes from China.

### Haplotype networks and phylogenetic analyses

**Figure 2:**
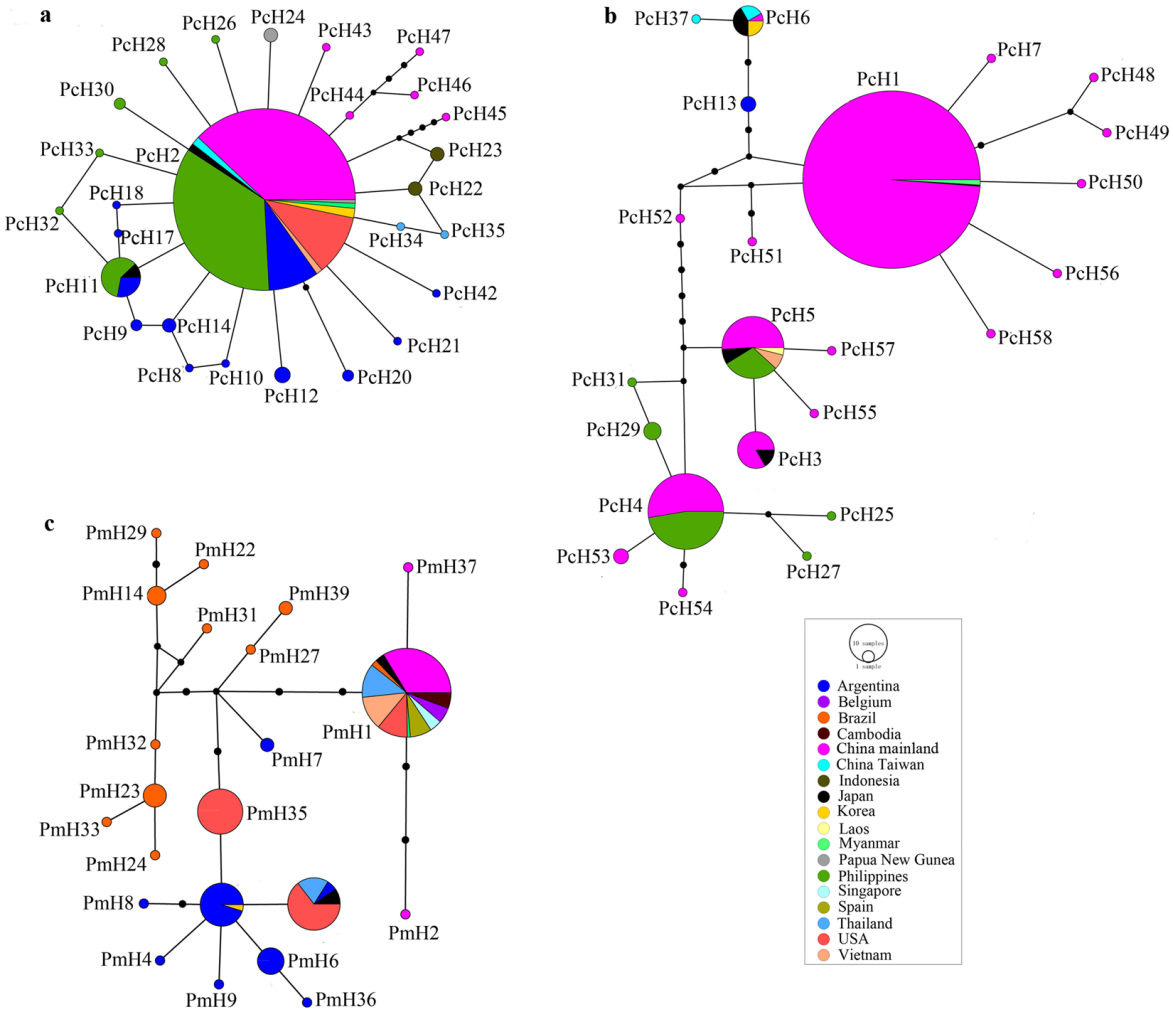
COI haplotype networks reconstructed using 95% connection limit both *P. canaliculata* and *P. maculata*. (**a**) Network A and (**b**) Network B represent for *P. canaliculata*, respectively. (**c**) Network C represents for *P. maculata*. They are the three networks containing haplotypes present in mainland China. The colors indicate haplotypes from different countries. For each haplotype, the size of the circle is proportional to the observed frequencies. PcH and PmH represent haplotypes for *P. canaliculata* and *P. maculata*, respectively.

Under a 95% parsimony limit, haplotype analyses produced three independent networks for *P. canaliculata* and seven separate networks for *P. maculata*. Only two of the *P. canaliculata* networks and one of the *P. maculata* network included haplotypes from mainland China (Fig. 2a,b, and c). Networks for both *P*. *canaliculata* and *P. maculata* corresponded to well supported (BS values ≥ 95%) clades in the ML tree.

For *P. canaliculata*, the 24 haplotypes from China occurred in two of the networks (Network A and Network B), with 209 sequences representing six haplotypes (PcH2 and PcH43~ PcH47) in Network A and 509 sequences representing 18 haplotypes (PcH1, PcH3~PcH7, and PcH48~ PcH58) in Network B (Fig. 2a,b). The third network contained eight haplotypes, including six unique to Argentina, one unique to Uruguay, and one shared by Taiwan and Japan (Fig. S2).

For Network A, PcH2 was the only shared haplotype out of the six detected haplotypes in China. It shared among populations found in Argentina (native) and non-native ranges, including China, the Philippines, Japan, Korea, Vietnam, Myanmar, USA, and Papua New Guinea (non-native) (Fig. 2a). The remaining 25 unique haplotypes and one shared haplotype (Argentina, Japan and the Philippines) were one to five steps away from PcH2, creating a star-like structure which indicated that PcH2 was an founding haplotypes (Fig. 2a). Among 23 haplotypes in Network B, PcH13 was the only one found in Argentina and 18 haplotypes were found in China (Fig. 2b). Five haplotypes (PcH1, PcH3~PcH6) detected in China were shared by snails found in Asian countries; one haplotype unique to Taiwan and the remaining 12 haplotypes were unique to mainland China (Fig. 2b).

For *P. maculata*, one (Network C) of seven networks contained haplotypes detected in China (Fig. 2c). The other six networks contained 15 haplotypes representing 39 sequences sampled from their native range in Brazil (Fig. S3). In Network C, PmH1 was the only haplotypes shared by snails found in Brazil and China, and also other non-native countries, including Japan, Vietnam, Thailand, Cambodia, Singapore, USA, Spain, and Belgium. PmH3 and PmH5 were the shared haplotypes found in Argentina (native range) and non-native ranges from USA, Thailand, Japan or Korea (Fig. 2c).

### Mismatch distribution

**Figure 3:**
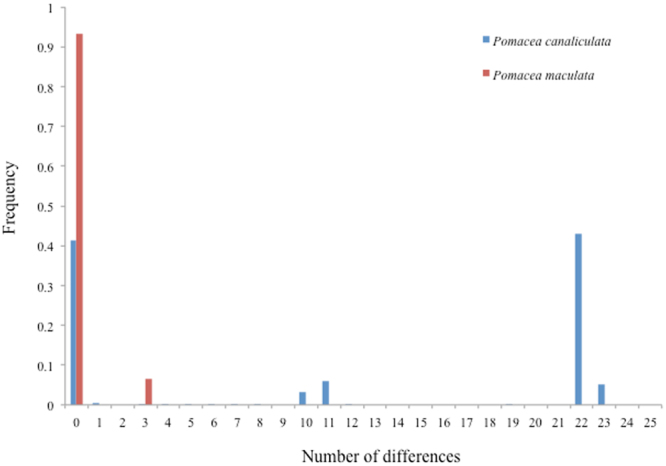
Mismatch distributions of *P. canaliculata* (blue) and *P. maculata* (red) sequences from China. The major peaks of samples in both distributions correspond to the haplotype groups recovered with network analysis (Fig. 2).

The mismatch distribution for Chinese *P. canaliculata* sequences produced two distinct and well separated peaks, which exhibited high frequencies of number of nucleotide differences, with the intermediate peak representing single rare introduced samples (Fig. 3). However, the mismatch distribution for Chinese *P. maculata* sequences produced a single major peak (Fig. 3).

## Discussion

China is the world’s fourth-largest country in terms of landmass, and its highly diverse topography and climate provides numerous opportunities for non-native species to find suitable habitats, establish, and potentially become invasive pests^11^. There are 560 confirmed invasive alien species in China, resulting in an estimated annual economic loss of more than US$18.9 billion^30,31^. The surge in economic growth following the implementation of the landmark Reform and Opening in 1978 was a milestone in China’s national policy and economic development, but resulted in the 1980s and 1990s in the introduction and spread of large numbers of invasive species^11^. Apple snails were one of the pests introduced and spread rapidly during this period.

The agricultural and environmental impacts, and associated economics costs of introduced species have led to a rising interest in studies on their ability to disperse, colonize, and establish in novel habitats^32,33^. Apple snails have colonized a wide range of aquatic systems in China, including rivers, paddies, pools, and ponds. The irrigated rice and wild rice (*Zizania latifolia*) ecosystems in southern areas provide an ideal environment for the dispersal and growth of the snails. Although the species of apple snails introduced to Asia and their origins have been elucidated^3^, their current distributions and origins in China have been less well understood.

Generally, ancestral populations possess higher levels of gene diversity than more recently established populations, which often display low diversity and few haplotypes^34^. The low haplotype diversity may be attributed either to the founder effect, such that invasive populations experience bottlenecks and genetic drift^19,35^, or to the bridge-head effect, in which the introduction of alien organisms to a non-native location may not be directly from the native range, but from a successful invasive population elsewhere^36^.

Both our and previous studies confirmed a much lower haplotype diversity of *P. maculata* in populations of China than in their native countries Argentina and Brazil with a statistic ratio of 3: 34^3,13,18^. However, unlike non-native *P. canaliculata* populations in Hawaii with a single haplotype represented by sequences from 89 snails^37^, snails in China possess higher haplotype diversity than in native populations. We found 25 haplotypes from Chinese populations of *P. canaliculata*, thus five more than that from both Argentina and Uruguay (Table 2). It was indicated that apple snail populations had admixed in the course of invasion in China^18^. These admixed populations support the conclusion by Hayes *et al*. of multiple source introductions initially out of South America^3^. Such introduction scenarios increase genetic diversity of introduced populations over that of a single source introduction, thus possibly facilitating the establishment despite a bottleneck^38^. However, there were 14 haplotypes from Chinese populations of *P. canaliculata* reported by Lv *et al*., and not recovered in any other study or shared with any other countries. Since unique mutations were carefully checked and ambiguous bases were confirmed by Lv *et al*.^13^, the most parsimonious explanation is that these unique haplotypes come from unsampled populations in the native range. Two explanations are possible for the discrepancy in haplotype diversity between the previous study and this one for *P. canaliculata*: (1) the larger sample size of Lv *et al*. and the less extensive sampling in other countries for this study^13^, and/or 2) apple snails in China may have lost haplotypes as following a bottleneck^39^. However, further genetic analysis is needed to clarify this in any situation.

Haplotype diversity and distributions revealed consistent patterns with which revealed by Hayes *et al*. that Chinese populations of *P. canaliculata* shared an Argentinian origin with other introduced apple snails in Asia and experienced multiple introductions^3^. Different from *P. canaliculata*, Hayes *et al*. also indicated two introduction lineages of non-native *P. maculata* from Brazil and Argentina independently^3^. However, just single lineage of *P. maculata* from Brazil was introduced into and established in China. The presence of diverse shared haplotypes among different populations from different countries indicated a complicated pattern of introduction into China and other non-native countries.

According to early accounts, driven by the commercial benefits of aquaculture, apple snails were introduced to national wide including cities in both southern and northern China, like Beijing, Tianjin, and Liaoning province^12^. Our study of the current distribution of apple snails in China revealed that apple snails have established natural populations in most of southern China but none in north area. We found natural populations of apple snails in north area of Zhejiang provinces (longitude 30.31°N) and south area of Jiangsu provinces (longitude 31.23°N). Comparing with the sampling sites in previous studies^13,18,40^, our data indicated that apple snails tended to expand into northern China. In addtion,We discovered a new *P. maculata* population in Zhejiang province, which is ~1876 km far from the reported *P. maculata* populations in Sichuan and Chongqing basin, indicating an invisible or unobtrusive spread of apple snails.

It is recorded that apple snails were first introduced into Asia via Taiwan in 1979, and then introduced to other Asian countries, including Japan and the Philippines^9,41,42^. Subsequently, the prevalence of snails-farming and frequent agriculture contacts among our neighbor countries made a round introduction of apple snails and speed the wide spread of apple snails in Asia^43,44^. Nevertheless the native origins of invasive apple snails were explicated, such complicated pattern in introduced ranges was probably result from extensive influence by human activities. Human factors were also the most likely driver for the fast spread of apple snails in China. Our study for understanding the origin and distribution of apple snails is important for early detection and control of these invasive snails to slow the rate of new invasions in China.

## Electronic supplementary material

Supplement Material

## References

[1] Hayes KA (2015). Insight from an integrated view of the biology of apple snails (Caenogastropoda: Ampullariidae). Malacologia.

[2] Rawlings TA, Hayes KA, Cowie RH, Collins TM (2007). The identity, distribution, and impacts of non–native apple snails in the continental United States. BMC Evol. Biol..

[3] Hayes KA, Joshi RC, Thiengo SC, Cowie RH (2008). Out of South America: multiple origins of non-native apple snails in Asia. Divers. Distrib..

[4] Carlsson NOL, Bronmark C, Hansson LA (2004). Invading herbivory: the golden apple snail alters ecosystem functioning in Asian wetlands. Ecology.

[5] Qiu JW, Chan MT, Kwong KL, Sun J (2011). Consumption, survival and growth in the invasive freshwater snail *Pomacea canaliculata*: does food freshness matter?. J Mollus. Stud..

[6] Barnes MA, Fordham RK, Burks RL, Hand JJ (2008). Fecundity of the exotic apple snail, *Pomacea insularum*. J N. Am. Benthol. Soc..

[7] Havel JE, Bruckerhoff LA, Funkhouser MA, Gemberling AR (2014). Resistance to desiccation in aquatic invasive snails and implications for their overland dispersal. Hydrobiologia.

[8] Kim YS, Choi KC (2014). 215 snail and slug, markers of epithelial mesenchymal transition, appeared to be altered by alkyl-phenols, bisphenol a and nonyl-phenol, in ovarian cancer cells expressing estrogen receptors. Reprod. Fert. Develop..

[9] Mochida O (1991). Spread of freshwater *Pomacea* snails (Pilidae, Mollusca) from Argentina to Asia. Micronesica.

[10] Lv S (2009). Invasive snails and an emerging infectious disease: results from the first national survey on *Angiostrongylus cantonensis* in China. PLoS Neglect. Trop. D..

[11] Wan FH, Yang NW (2016). Invasion and management of agricultural alien insects in China. Annu. Rev. Entomol..

[12] Yang Y (2010). Historical invasion, expansion process and harm investigation of *Pomacea canaliculata* in China. Chinese Agri. Sci. Bull..

[13] Lv S (2013). Phylogenetic evidence for multiple and secondary introductions of invasive snails: *Pomacea* species in the People’s Republic of China. Divers. Distrib..

[14] Hayes KA, Cowie RH, Thiengo SC, Strong EE (2012). Comparing apples with apples: clarifying the identities of two highly invasive Neotropical Ampullariidae (Caenogastropoda). Zool. J Linn. Soc..

[15] Cazzaniga NJ (2002). Old species and new concepts in the taxonomy of *Pomacea* (Gastropoda: Ampullariidae). Biocell.

[16] Cowie, R. H., Hayes K. A. & Thiengo, S. C. What are apple snails? Confused taxonomy and some preliminary resolution in *Global advances in ecology and management of golden apple snails* (eds Joshi, R. C. & Sebastian, L. S.) 3–24 (Philippine Rice Research Institute, Philippines, 2006).

[17] Lowe, S., Browne, M., Boudjelas, S. & DePoorter, M. 100 of the world’s worst invasive alien species, a selection from the global invasive species database. Published by The Invasive Species Specialist Group (ISSG) a specialist group of the Species Survival Commission (SSC) of theWorld Conservation Union (IUCN), 12 pp. First published as special lift-out in Aliens 12 December 2000. Updated andreprinted version: November 2004.

[18] Song HM (2010). Sequencing cytochrome oxidase subunit I of mitochondrial DNA and the taxonomic status of apple snails. Chinese J. Zool..

[19] Matsukura K, Okuda M, Cazzaniga NJ, Wada T (2013). Genetic exchange between two freshwater apple snails, *Pomacea canaliculata* and *Pomacea maculata* invading East and Southeast Asia. Biol. Invasions.

[20] Folmer O, Black M, Hoeh W, Lutz R, Vrijenhoek R (1994). DNA primers for amplification of mitochondrial cytochrome c oxidase subunit I from diverse metazoan invertebrates. Mol. Mar. Biol. Biotech..

[21] Goodstadt L, Ponting CP (2001). CHROMA: consensus-based colouring of multiple alignments for publication. Bioinformatics.

[22] Hayes KA, Cowie RH, Thiengo SC (2009). A global phylogeny of apple snails: Gondwanan origin, genetic relationships, and the influence of outgroup choice (Caenogastropoda: Ampullariidae). Biol. J Linn. Soc..

[23] Tamura K, Stecher G, Peterson D, Filipski A, Kumar S (2013). MEGA6: Molecular evolutionary genetics analysis version 6.0. Mol. Biol. Evol..

[24] Darriba D, Taboada GL, Doallo R, Posada D (2012). jModelTest 2: more models, new heuristics and parallel computing. Nat Methods.

[25] Felsenstein J (1985). Confidence limits on phylogenies: an approach using the bootstrap. Evolution.

[26] Clement M, Posada D, Crandall KA (2000). TCS: a computer program to estimate gene genealogies. Mol. Ecol..

[27] Librado P, Rozas J (2009). DnaSPv5: a software for comprehensive analysis of DNA polymorphism data. Bioinformatics.

[28] Rogers A, Harpending H (1992). Population growth curves in the distribution of pairwise genetic differences. Mol. Biol. Evol..

[29] Harpending H (1994). Signature of ancient population growth in a low resolution mitochondrial DNA mismatch distribution. Hum. Biol..

[30] Xu HG (2012). Aninventory of invasive alien species in China. NeoBiota.

[31] Ding, H., Li, M. Y. & Xu, H. G. Assessing economic costs of invasive exotic species in China in *Alien Species Invasion, Biosafety and Genetic Resources* (eds Xu, H. G., Wang, J. M., Qiang, S. & Wang, C. Y.) 78–128 (Beijing: Science, (2004).

[32] Cote J, Fogarty S, Weinersmith K, Brodin T, Sih A (2010). Personality traits and dispersal tendency in the invasive mosquitofish (*Gambusia affinis*). Proc. Biol. Sci..

[33] Sabour B (2013). *Sargassum muticum* (Yendo) Fensholt (Fucales, Phaeophyta) in Morocco, an invasive marine species new to the Atlantic coast ofAfrica. Aquat. Invaions..

[34] Wu Y, Mcpheron BA, Wu JJ, Li ZH (2012). Genetic relationship of the melon fly, *Bactrocera cucurbitae*, (Diptera: Tephritidae) inferred from mitochondrial DNA. Insect Sci..

[35] Shirk RY, Hamrick JL, Zhang C, Qiang S (2014). Patterns of genetic diversity reveal multiple introductions and recurrent founder effects during range expansion in invasive populations of *Geranium carolinianum* (Geraniaceae). Heredity.

[36] Lombaert E (2010). Bridgehead effect in the worldwide invasion of the biocontrol harlequin ladybird. PLoS One.

[37] Tran CT, Hayes KA, Cowie RH (2008). Lack of mitochondrial DNA diversity in invasive apple snails (Ampullariidae) in Hawaii. Malacologia.

[38] Simon A (2011). Invasive cyprinid fish in Europe originate from the single introduction of an admixed source population followed by a complex pattern of spread. PLoS One.

[39] Nei M, Maruyama T, Chakraborty R (1975). The bottleneck effect and genetic variability in populations. Evolution.

[40] Lv S (2011). The emergence of angiostrongyliasis in the People’s Republic of China: the interplay between invasive snails, climate change and transmission dynamics. Freshwater Biol..

[41] Naylor R (1996). Invasions in agriculture: assessing the cost of the golden apple snail in Asia. Ambio.

[42] Joshi RC, Sebastian LS (2006). Global advances in the ecology and management of golden apple snails.

[43] Cowie RH, Barker GM (2002). Apple snails (Ampullariidae) as agricultural pests: their biology, impacts and management. Molluscs as crop pests.

[44] Yusa Y, Sugiura N, Wada T (2006). Predatory potential of freshwater animals on an invasive agricultural pest, the apple snail *Pomacea canaliculata* (Gastropoda: Ampullariidae), in southern Japan. Biol. Invasions.

