# Supplementary material for: Distribution and the origin of invasive apple snails, Pomacea canaliculata and P. maculata (Gastropoda: Ampullariidae) in China
Source: Sci Rep. 2018 Jan 19;8:1185. doi: 10.1038/s41598-017-19000-7 (PMC5775418; doi:10.1038/s41598-017-19000-7)
Supplement: Supplementary file 1 — Supplement Material [file 41598_2017_19000_MOESM1_ESM.pdf]

Distribution and the origin of invasive apple snails, *Pomacea canaliculata* and *P. maculata* (Gastropoda: Ampullariidae) in China

Qian-Qian Yang[§], Su-Wen Liu[§], Chao He, Xiao-Ping Yu[*]

Zhejiang Provincial Key Laboratory of Biometrology and Inspection and Quarantine, College of Life Science, China Jiliang University, Hangzhou 310018, China

[§] These authors contributed equal to this work.

[*]Corresponding author: Xiao-Ping Yu, E-mail: yxp@cjlu.edu.cn; Tel: +86 571 86836006; Fax: +86 571 86836079

Email addresses:

Q.-Q.Y.: yqq@cjlu.edu.cn

S.-W. L.: 1418677249@qq.com

C.H.: 545731107@qq.com

Supplement Material Appendix

Table S1 Available COI sequences in GenBank used in this study.

Access No.	Species	Isolate	Country
JX845573	*Pomacea maculata*	-	USA
GU133205	*Pomacea maculata*	Pom2	Spain
GU133206	*Pomacea maculata*	Pom3	Spain
GU133207	*Pomacea maculata*	Pom9	Spain
GU236486	*Pomacea maculata*	Ebro16	Spain
GU236489	*Pomacea maculata*	Ebro19	Spain
GU236490	*Pomacea maculata*	Ebro20	Spain
GU236491	*Pomacea maculata*	Ebro21	Spain
AB433757	*Pomacea canaliculata*	Kitaura_01	Japan
AB433758	*Pomacea canaliculata*	Iwata_01	Japan
AB433759	*Pomacea canaliculata*	Nara_01	Japan
AB433760	*Pomacea canaliculata*	Nara_02	Japan
AB433761	*Pomacea canaliculata*	Kakogawa_01	Japan
AB433762	*Pomacea canaliculata*	Yurihama_01	Japan
AB433763	*Pomacea canaliculata*	Yurihama_02	Japan
AB433764	*Pomacea canaliculata*	Fukuyama_01	Japan
AB433765	*Pomacea canaliculata*	Matsuyama_01	Japan
AB433766	*Pomacea canaliculata*	Matsuyama_02	Japan
AB433767	*Pomacea canaliculata*	Kamimine_01	Japan
AB433768	*Pomacea canaliculata*	Kikuchi_01	Japan
AB433769	*Pomacea canaliculata*	Kikuchi_02	Japan
AB433770	*Pomacea canaliculata*	Kikuchi_03	Japan
AB433771	*Pomacea canaliculata*	Kikuchi_04	Japan
AB433772	*Pomacea canaliculata*	Iheya_01	Japan
AB433773	*Pomacea canaliculata*	Nago_01	Japan
AB433774	*Pomacea canaliculata*	IshigakiW_01	Japan
AB433775	*Pomacea canaliculata*	IshigakiPo_01	Japan
AB433776	*Pomacea maculata*	Iwata_01	Japan
AB433777	*Pomacea maculata*	Fukuyama_01	Japan
AB433778	*Pomacea maculata*	IshigakiW_01	Japan
AB433779	*Pomacea maculata*	IshigakiPo_01	Japan
AB433780	*Pomacea maculata*	IriomotePo_01	Japan
AB433781	*Pomacea maculata*	IriomotePa_01	Japan
AB728574	*Pomacea canaliculata*	Hap1	Argentina
AB728575	*Pomacea canaliculata*	Hap2	Argentina
AB728576	*Pomacea canaliculata*	Hap3	Argentina
AB728577	*Pomacea canaliculata*	Hap4	Argentina

AB728578	*Pomacea canaliculata*	Hap5	Argentina
AB728585	*Pomacea canaliculata*	Hap1	Argentina
AB728586	*Pomacea canaliculata*	Hap2	Argentina
AB728587	*Pomacea canaliculata*	Hap3	Argentina
AB728588	*Pomacea canaliculata*	Hap4	Argentina
AB728579	*Pomacea maculata*	Hap1	Argentina
AB728580	*Pomacea maculata*	Hap2	Argentina
AB728582	*Pomacea maculata*	Hap1	Argentina
AB728583	*Pomacea maculata*	Hap2	Argentina
AB728584	*Pomacea maculata*	Hap3	Argentina
EF514967	*Pomacea canaliculata*	HI_096	USA
EF514968	*Pomacea canaliculata*	HI_095	USA
EF514969	*Pomacea canaliculata*	HI_097	USA
EF514970	*Pomacea canaliculata*	AZ_087	USA
EF514971	*Pomacea canaliculata*	AZ_088	USA
EF514972	*Pomacea canaliculata*	AZ_086	USA
EF514973	*Pomacea canaliculata*	AZ_089	USA
EF514974	*Pomacea canaliculata*	AZ_090	USA
EF514975	*Pomacea canaliculata*	AZ_091	USA
EF514976	*Pomacea canaliculata*	AZ_092	USA
EF514977	*Pomacea canaliculata*	AZ_093	USA
EF514978	*Pomacea canaliculata*	AZ_094	USA
EF514979	*Pomacea canaliculata*	CA_080	USA
EF514980	*Pomacea canaliculata*	CA_081	USA
EF514981	*Pomacea canaliculata*	CA_082	USA
EF514982	*Pomacea canaliculata*	CA_083	USA
EF514983	*Pomacea canaliculata*	CA_084	USA
EF514984	*Pomacea canaliculata*	CA_085	USA
EF514942	*Pomacea maculata*	FL_001	USA
EF514943	*Pomacea maculata*	FL_002	USA
EF514944	*Pomacea maculata*	FL_003	USA
EF514985	*Pomacea maculata*	FL_004	USA
EF515013	*Pomacea maculata*	TX_042	USA
EF515014	*Pomacea maculata*	TX_043	USA
EF515015	*Pomacea maculata*	TX_044	USA
EF515016	*Pomacea maculata*	TX_045	USA
EF515020	*Pomacea maculata*	TX_039	USA
EF515022	*Pomacea maculata*	TX_049	USA
EF515023	*Pomacea maculata*	TX_050	USA
EF515024	*Pomacea maculata*	TX_040	USA
EF515025	*Pomacea maculata*	TX_041	USA
EF515026	*Pomacea maculata*	FL_031	USA

EF515027	*Pomacea maculata*	FL_032	USA
EF515028	*Pomacea maculata*	FL_030	USA
EF515029	*Pomacea maculata*	FL_033	USA
EF515030	*Pomacea maculata*	FL_034	USA
EF515031	*Pomacea maculata*	FL_005	USA
EF515032	*Pomacea maculata*	FL_006	USA
EF515033	*Pomacea maculata*	FL_007	USA
EF515034	*Pomacea maculata*	FL_008	USA
EF515035	*Pomacea maculata*	FL_009	USA
EF515036	*Pomacea maculata*	FL_010	USA
EF515037	*Pomacea maculata*	FL_011	USA
EF515038	*Pomacea maculata*	FL_012	USA
EF515039	*Pomacea maculata*	FL_013	USA
EF515040	*Pomacea maculata*	FL_014	USA
EF515041	*Pomacea maculata*	FL_015	USA
EF515042	*Pomacea maculata*	FL_020	USA
EF515043	*Pomacea maculata*	FL_021	USA
EF515044	*Pomacea maculata*	FL_022	USA
EF515045	*Pomacea maculata*	FL_023	USA
EF515046	*Pomacea maculata*	FL_024	USA
EF515047	*Pomacea maculata*	FL_025	USA
EF515048	*Pomacea maculata*	FL_016	USA
EF515049	*Pomacea maculata*	FL_017	USA
EF515050	*Pomacea maculata*	FL_026	USA
EF515051	*Pomacea maculata*	FL_027	USA
EF515052	*Pomacea maculata*	FL_028	USA
EF515053	*Pomacea maculata*	FL_029	USA
EF515054	*Pomacea maculata*	GA_035	USA
EF515055	*Pomacea maculata*	GA_036	USA
EF515056	*Pomacea maculata*	GA_037	USA
EF515057	*Pomacea maculata*	FL_018	USA
EF515058	*Pomacea maculata*	FL_019	USA
EU523129	*Pomacea canaliculata*	HI39	USA
FJ946821	*Pomacea canaliculata*	JX_03	China
FJ946822	*Pomacea canaliculata*	QZ_03	China
FJ946823	*Pomacea canaliculata*	MM_01	China
FJ946824	*Pomacea canaliculata*	GZ_01	China
FJ946825	*Pomacea canaliculata*	QZ_01	China
FJ946828	*Pomacea maculata*	SC_02	China

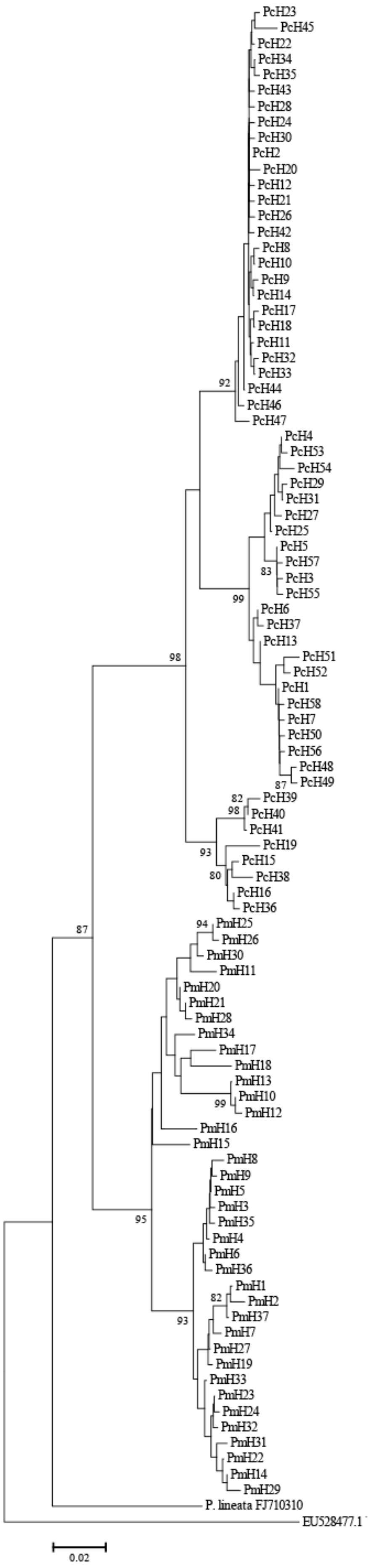
PcH23
PcH45
PcH22
PcH34
PcH35
PcH43
PcH28
PcH24
PcH30
PcH2
PcH20
PcH12
PcH21
PcH26
PcH42
PcH8
PcH10
PcH9
PcH14
PcH17
PcH18
PcH11
PcH32
PcH33
92
PcH44
PcH46
PcH47
PcH4
PcH53
PcH54
PcH29
PcH31
PcH27
PcH25
PcH5
PcH57
83
PcH3
PcH55
99
PcH6
PcH37
PcH13
98
PcH51
PcH52
PcH1
PcH58
PcH7
PcH50
PcH56
PcH48
87
PcH49
82
PcH39
98
PcH40
PcH41
93
PcH19
PcH15
80
PcH38
PcH16
PcH36
87
94
PmH25
PmH26
PmH30
PmH11
PmH20
PmH21
PmH28
PmH34
PmH17
PmH18
PmH13
99
PmH10
PmH12
PmH16
PmH15
PmH8
PmH9
PmH5
PmH3
95
PmH35
PmH4
PmH6
PmH36
PmH1
82
PmH2
PmH37
93
PmH7
PmH27
PmH19
PmH33
PmH23
PmH24
PmH32
PmH31
PmH22
PmH14
PmH29
P. lineata FJ710310
EU528477.1
0.02

Figure S1 Maximum likelihood phylogenetic tree of the haplotypes generated from the 1464 COI sequences used in this study. PcH represents haplotypes of *P. canaliculata,* PmH represents haplotypes of *P. maculata.*

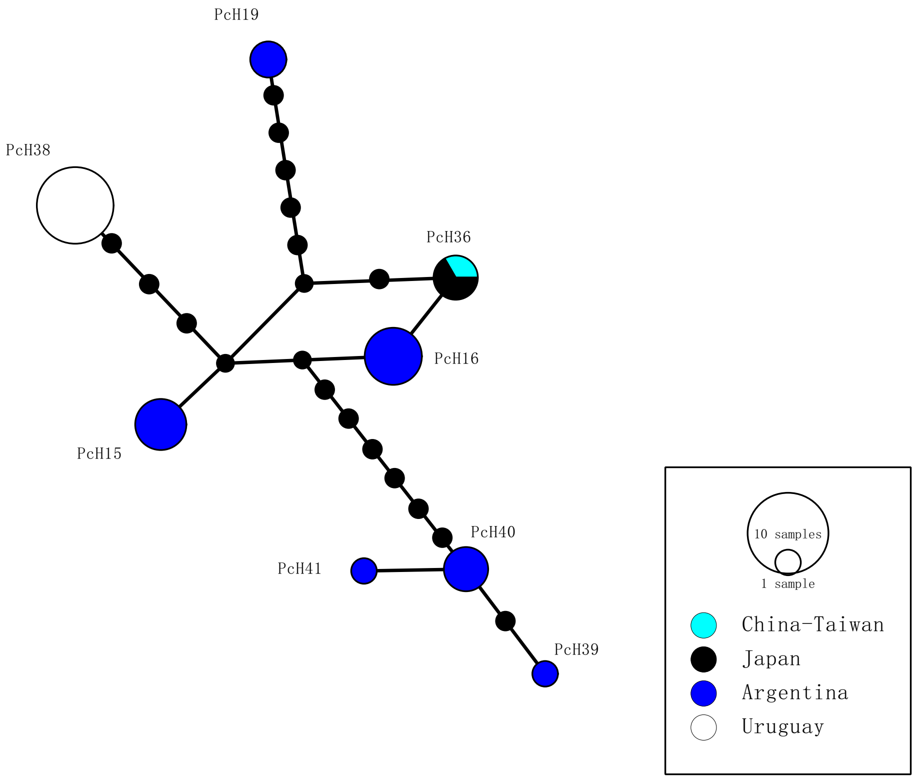

Figure S2 The third network for *P. canaliculata*. The colors indicate haplotypes from different countries. For each haplotype, the size of the circle is proportional to the observed frequencies. PcH represents haplotypes for *P. canaliculata*.

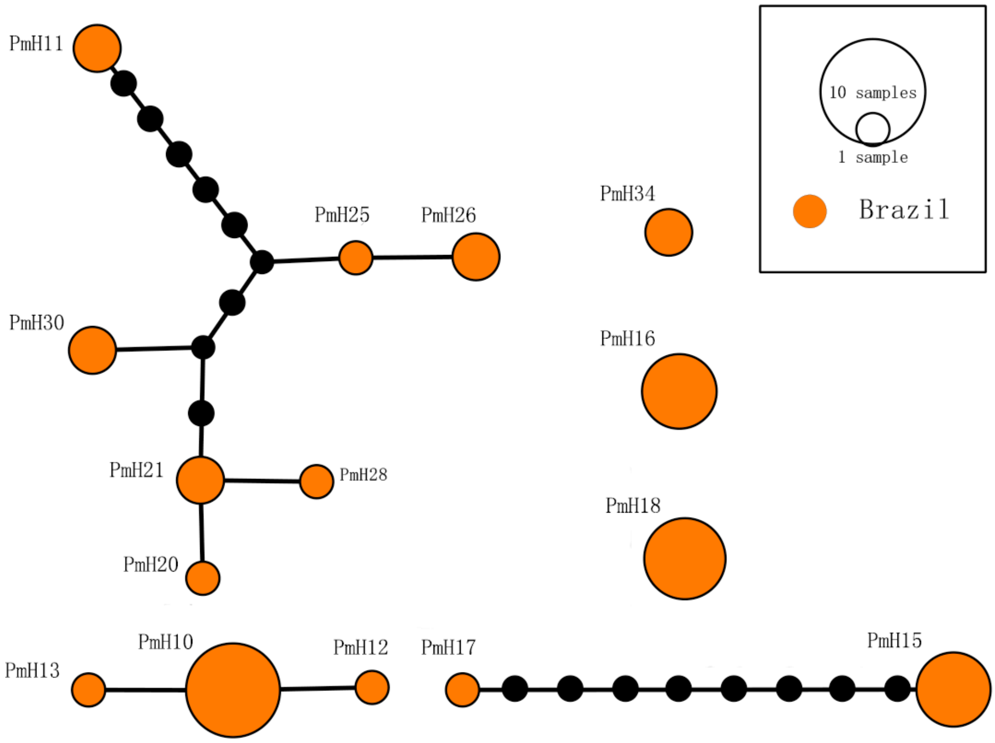

Figure S3 The six networks for *P. maculata* representing sequences sampled from Brazil. For each haplotype, the size of the circle is proportional to the observed frequencies. PmH represents haplotypes for *P. maculata*.
